# Effect of surface preparation with Nd:YAG and Er,Cr:YSGG lasers on the repair bond strength of lithium disilicate glass ceramic to a silorane-based composite resin

**DOI:** 10.15171/joddd.2018.003

**Published:** 2018-03-14

**Authors:** Mohammad Esmaeel Ebrahimi Chaharom, Fatemeh Pournaghi Azar, Narmin Mohammadi, Rezvan Nasiri

**Affiliations:** ^1^Department of Operative Dentistry, Faculty of Dentistry, Tabriz University of Medical Sciences, Tabriz, Iran; ^2^Department of Operative Dentistry, Faculty of Dentistry, Arak University of Medical Sciences, Arak, Iran

**Keywords:** Er, Cr:YSGG laser, Nd:YAG laser, HF, lithium disilicate glass ceramic

## Abstract

***Background.*** This study was undertaken to evaluate the repair bond strength of lithium disilicate glass ceramic to a silorane-based composite resin after surface preparation with Nd:YAG and Er,Cr:YSGG lasers.

***Methods.*** A total of 102 lithium disilicate glass ceramic samples (IPS e.max Press), measuring 5 mm in diameter and 4 mm in thickness, were randomly assigned to 6 groups (n=17): group 1, no surface preparation (control); group 2, acid etching with 9.5% hydrofluoric acid (HF); group 3, surface preparation with 4.5-W Nd:YAG laser; group 4, surface preparation with 6-W Nd:YAG laser; group 5, surface preparation with 1.5-W Er,Cr:YSGG laser; and group 6, surface preparation with 6-W Er,Cr:YSGG laser. After preparation of surfaces and application of silane, all the samples were repaired with the use of a silorane-based composite resin, followed by storage in distilled water at a temperature of 37°C for 24 hours and thermocycling. Finally, the samples were subjected to a shearing bond strength test; the fracture modes were determined under a stereomi-croscope.

***Results.*** There were significant differences between the HF group and the other groups (P=0.000). Two-by-two comparisons of the other groups revealed no significant differences (P>0.05).

***Conclusion.*** Use of HF proved the most effective surface preparation technique to increase the repair bond strength between lithium disilicate glass ceramic and silorane-based composite resin; compared to the control group.

## Introdoction

In recent years there has been an ever-increasing interest in the use of inlays, onlays and ceramic laminates due to their high esthetic appearance.^1^ However, all-ceramic restorations might undergo failure due to fractures, cracks and chipping resulting from their brittle nature and structural defects.^2^ It is not always a favorable choice to replace fractured ceramic restorations because the tooth structure is compromised during replacement of the restoration and more costs are inflicted on the patients.^3^ One of the corrective methods for fractured porcelain restorations is to repair them directly with composite resins.^4,5^

Lithium disilicate glass ceramic is a new ceramic with high strength, which is used to fabricate full crowns and very thin veneers.^6^ Different techniques are used to achieve a bond with lithium disilicate glass ceramic, with advantages of chemical bonds and mechanical retention at ceramic‒resin interface.^7^ When etching is carried out with HF and silane is applied, the bond strength of composite resin to different ceramics increases significantly.^8^ However, etching with HF is dangerous for the patient and the dentist, and HF should be completely removed before the bonding procedure. Recently, use of laser has been suggested as an easy and safe technique for preparation of dental material surfaces.^1^

In different studies different laser types have been used to prepare porcelain surfaces.^9-12^ Gokce et al (2007) carried out a study on the bond strength of lithium-based ceramics with the use of acid etching with HF and Er:YAG laser beams for surface preparation. The results showed that use of laser with a power setting of 300 mJ and acid etching increased the shear bond strength values and an increase in laser power setting resulted in a decrease in shear bond strength values.^13^ Another study showed that the micromorphology of the surface of lithium disilicate glass ceramic after surface preparation with Nd:YAG laser beams was similar to that of unprepared ceramic surfaces. The study showed that the micromorphology of the surface of lithium disilicate glass ceramic after surface preparation with Nd:YAG laser was similar to that of unprepared ceramic surfaces. The study showed that acid etching with HF is necessary to achieve higher shear bond strength.^14^

This study was undertaken to evaluate the effect of preparation of ceramic surfaces with Nd:YAG and Er,Cr:YSGG laser beams and the repair bond strength of lithium disilicate glass ceramic to a silorane-based composite resin.

## Methods

A total of 102 IPS e.max Press lithium disilicate glass ceramic disks (Ivoclar Vivadent, Schaan, Liechtenstein), measuring 5 mm in diameter and 4 mm in height, were prepared in the porcelain curing oven using the wax elimination technique following manufacturer’s instructions. No glazing procedures were carried out on sample surfaces. The samples were mounted in auto-curing acrylic resin (Acropars, Iran). After the samples were cured and prepared, their surfaces were smoothed and polished with 600- and 1000-grit silicon carbide disks (Carbimet Paper Disc, Buehler, Lake Forest, IL, USA) under water cooling. The sample surfaces were rinsed with water to remove contaminations before surface preparation and air-dried. At this stage the samples were randomly assigned to 6 groups (n=17) as follows:

Group 1: No surface preparation was carried out in this group (control).

Group 2: The sample surfaces were etched with 95% HF (Porcelain Etch, Ultradent Product, Inc. South Jordan, UT, USA) for 60 seconds and rinsed for 5 seconds to remove all the residual acid. The samples were finally air-dried.

Group 3: The surfaces of the ceramic samples were prepared with Nd:YAG laser beams (Nd:YAG Dental Laser, LAMADA Scientifica, Srl, Vicenza, Italy), under air/water spray, with the following laser specifications: energy parameter = 30 mJ, power setting = 4.5 W, repetition rate = 15 Hz and wavelength = 1.064 μm. The laser conducting tip was placed perpendicular to the ceramic surface at a distance of 1 mm from the surface for 60 seconds. Then the samples were rinsed for 180 seconds and dried for 15 seconds.

Group 4: All the procedures were similar to those in groups 3, except that Nd:YAG laser was used for surface preparation with the following laser specifications: power setting = 6 W, energy parameter = 300 mJ, repetition rate = 20 Hz and wavelength = 1.064 μm.

Group 5: The ceramic surfaces were prepared with Er,Cr:YSGG laser irradiation (Millenium, biolase technology, inc,san clement,CA,USA ) with the following laser specifications under air/water spray: power setting = 1.5 W, wavelength = 2.78 μm and energy parameter = 300 mJ. The laser conducting tip was placed perpendicular to the sample surfaces 1 mm away from the surface for 60 seconds. Then the samples were rinsed for 180 seconds and dried for 15 seconds.

Group 6: All the ceramic surface preparation procedures were similar to those in group 5 except that Er,Cr:YSGG laser was used with the following laser specifications: power setting = 6 W, energy parameter = 300 mJ and wavelength = 2.78 nm.

At this stage, silane (Porcelain Silane, Ultradent Product, Inc. South Jordan, UT, USA) was applied to all the dry sample surfaces for 60 seconds and dried with an air stream. Subsequently, the primer and adhesive agent of the self-etch silorane system (Silorane System Adhesive, 3M, ESPE, Dental Product, St, Paul, Mn, USA) were applied using the manufacturer’s instructions. After curing of the bonding agent for 20 seconds and placement of plastic molds measuring 3 mm in length and 3 mm in diameter at the center of each sample, P90 silorane-based composite resin (3M ESPE, Dental Product, St, Paul, Mn, USA) was placed on the porcelain surfaces using the incremental technique and light-cured for 40 seconds with Astralis 7 light-curing unit (Astralis 7, Ivoclar Vivadent, Lichtenstein). After removal of the plastic molds, the samples were light-cured once again for 20 seconds. The samples were immersed in distilled water at 37ºC for 24 hours and underwent a 500-round thermocycling procedure with a dwell time of 30 seconds and a transfer time of 10 seconds.^15^

Then the samples mounted in acrylic resin molds underwent a shearing force at a strain rate of 1 mm/min by the chisel-shaped blade placed at the fractured porcelain‒composite resin interface in a Hounsfield test equipment (H5K-S model, England) to measure the repair bond strength of lithium disilicate glass ceramic to silorane-based composite resin.

The fracture patterns were determined under a stereomicroscope (Nikon, SMZ1500, America) at a magnification of **×**40 using the following classification:^7^

Adhesive failure: fracture at porcelain–composite resin interface

Cohesive failure: fracture within the porcelain or the composite resin

Mixed: a combination of the two above

ANOVA was used for the analysis of shear bond strength values, followed by post hoc Tukey tests at P<0.05.

## Results

**Figure 1:**
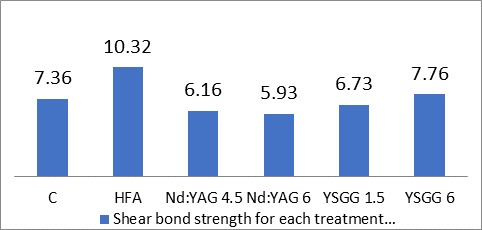
Mean shear bond strength values in each group. Values are shown as means + standard deviations for 17 samples in each group.

Table 1 and Figure 1 present the means, standard deviations and standard errors of shear bond strength values in different groups. One-way ANOVA revealed significant differences in shear bond strengths between the study groups (P=0.000). Two-by-two comparisons of the study groups with post hoc Tukey tests revealed significant differences in bond strengths between group 2 and the rest of the groups (P=0.000). In other cases, there were no significant two-by-two differences between the groups (P>0.05).

**Table 1:** Mean shear bond strength values, minimum (min), maximum (max) values, and standard deviations (SD) in MPa in each group

**Groups**	**Mean ± SD**	**M**in	**M**ax
**Control**	7.36±2.07^b^	3.86	11.63
**HF**	10.32±2.90^a^	5.78	14.85
**4.5-W Nd:YAG**	6.16±1.43^b^	4.42	9.04
**6-W Nd:YAG**	5.93±1.22^b^	3.82	8.91
**1.5-W Er,Cr:YSGG**	6.73±1.78^b^	4.09	11.62
**6-W Er,Cr:YSGG**	7.76±1.98^b^	5.57	12.05

^ab^Statistically different from each other (P<0.05).

### Analysis of fracture modes

Based on data presented in Table 2, the majority of the fractures were adhesive. Cohesive fracture was observed in 3 samples in the HF group (Table 2).

**Table 2:** Effect of various surface treatments on the frequency of failure modes after shear bond strength testing (n=17 per group)

**Groups**	**Type of rupture**		
	**Adhesive**	**Cohesive**	**Mixed**
**Control**	17	-	-
**HF**	13	3	1
**4.5-W Nd:YAG**	17	-	-
**6-W Nd:YAG**	17	-	-
**1.5-W YSGG**	17	-	-
**6-W YSGG**	17	-	-

## Discussion

The results of the present study showed that etching with 9.5% hydrofluoric acid is the most effective method for surface preparation of IPS e.max Press ceramic to achieve a stronger bond to a silorane-based composite resin. Several studies have advocated etching of the ceramic surface to increase the bond strength to composite resin when composite resin is used to repair fractures in ceramic restorations.^16,17^

Etching of porcelain and glass press ceramic surfaces with HF is an established and widely accepted technique to increase bond strength.^18-20^ This technique increases the surface roughness and surface area, resulting in an increase in physical reaction and an improvement in mechanical retention.^20,21^ It appears silorane is a necessary component for porcelain repair process because it modifies the superficial structure. Silorane brings about a stronger reaction with composite resin and can mediate a chemical bond between porcelain and composite resin.^22,23^ The silane modifies the surface layer of the substrate and forms a conversion layer,^24^ resulting in an increase in bond strength between the porcelain and composite resin.^25^ In the present study, the samples in the control group were smoothed and polished only with 600- and 1000-grit silicon carbide paper, followed by application of the silane on the ceramic surfaces. The low bond strength in this group shows that silane alone does not improve adhesion of the resin bonding adequately, consistent with the results of studies by Hayakawa et al and Shiu.^26,27^ However, Kamada et al^28^ reported higher bond strength in samples in which only 600-grit silicone paper and silane were used for surface preparation. Such a discrepancy in the results might be attributed to the use of 1000-grit silicon carbide paper and creation of a smoother surface with inadequate mechanical retention for the resin bonding in the present study.

Er:YAG, Nd:YAG and Er,Cr:YSGG laser beams have been used for surface conditioning of different dental materials.^9,29^ Several in vitro studies have evaluated the shear bond strength after preparation of the surface of ceramic restorations with different lasers and power settings. Akyil et al^30^ evaluated the repair shear bond strength vales of feldspathic ceramics with composite resin after preparation of surface with laser and reported lower bond strength with the use of 3-W Er:YAG laser and bond strength values with 1-W Nd:YAG laser, which was similar to that of unprepared surfaces. The results of this study showed that application of 6-W and 1.5-W Er,Cr:YSGG and 6-W and 4.5-W Nd:YAG lasers for surface preparation of IPS e.max Press ceramic did not increase the repair bond strength compared to the control group. In fact, the repair bond strength after application of Er,Cr:YSGG and Nd:YAG lasers was similar to and even less than that of unprepared surfaces, consistent with the results of a study by Akyil.^30^ In a study by Munir et al, preparation of the surface of lithium disilicate glass ceramic with 2-W Nd:YAG laser did not result in significant changes on the ceramic surface and the microscopic structure was similar to that of unprepared surfaces; however, IPS Empress 2 exhibited higher shear bond strength compared to IPS e.max Press.^14^ Such a discrepancy might be attributed to the nano-crystalline structure of IPS e.max Press, which is intended to increase the esthetic appearance. For this reason, we selected 4.5-W and 6-W output power for Nd:YAG laser irradiation that is higher than 2 W. In this context, Li^31^ reported that the bond strength decreased when the output power increased. Gocke^1^ and Pinar^13^ reported that shear bond strength decreased when Erbium laser was used with higher output power for surface treatment of IPS Empress 2 porcelain, too. The irregularities increased after higher output power laser applications, where the crystals were severely affected and dissociated. For further investigations, we used minimum and maximum output power of the Er,Cr:YSGG laser (1.5-W and 6-W).

Kara et al^32^ showed that surface roughness of IPS Empress 2 ceramic prepared with 10-W Er:YAG and 2-W Nd:YAG laser beams was comparable to that prepared with 5% HF. In another study, etching with 5% HF resulted in surface roughness comparable to that produced by Er:YAG laser in low-fusing ceramics.^33^ In addition, IPS Empress 2 exhibited a higher crystalloid content and higher bond strength compared to IPS Empress, irrespective of the type of surface preparation used.^34^ Albaky et al^35^ showed a higher flexural strength (400±40) in IPS e.max Press ceramic compared to IPS Empress and IPS Empress 2, which might the attributed to the higher needle-shaped crystal content of IPS e.max Press. Therefore, differences in the chemical compositions and microstructures of all-ceramic restorations can have an effect on the surface texture and the bond strength between ceramics and resin bonding agents. A decrease in the bond strength of ceramic surfaces prepared with laser might be attributed to inadequate microdepths created or to excessive destruction of the matrix phase or crystals or to the heat-damaged layer.^13^

It is important to analyze fracture modes in adhesion studies.^7^ Bond quality should not be evaluated only based on the assessment of bond strength data. Failure mode might provide important information about clinical limitations. In this study, there was no direct relationship between failure mode and bond strength results, as shown in Table 2. The most frequent fracture pattern was adhesive. Even in the HF group in which high bond strength was shown, the most frequent failure mode was adhesive, similar to that in the control group, indicating that use of silane on the ceramic surface resulted in the formation of hydrogen and prevalent bonds between the ceramic and the resin system.^35^ The results of the preset study showed that the bond strength after surface preparation with HF was higher than that after surface preparation with Nd:YAG and Er,Cr:YSGG laser and application of laser did not affect the bond strength. Since studies have shown the inherent risks of HF on biologic tissues,^37,38^ care should be exercised, including the use of rubber dams and etching gels, to prevent tissue injuries. Long-term clinical studies should be undertaken to evaluate the repair bond strength of composite resin to lithium disilicate glass ceramic. In addition, use of different parameters such as pulse duration, energy, density and power setting is necessary in future studies.

Some of the limitations of the present study included force loading, thermocycling, moisture and temperature conditions comparable to those prevailing in the oral cavity. A more accurate study with the use of SEM and energy dispersive spectrometer for debonded surfaces can yield more accurate data.

## Conclusion

The results of the present study on the repair bond strength of IPS e.max Press ceramic with application of HF and Nd:YAG and Er,Cr:YSGG laser beams showed that:

The highest repair bond strength values were recorded with the use of 9.5% hydrofluoric acid in comparison with those with the use of laser for surface preparation.

The repair bond strength values after surface preparation with laser were similar to those in the control group.

It appears the repair bond strength values achieved after surface preparation with Nd:YAG and Er,Cr:YSGG lasers with the laser specifications used in the present study are not adequate from a clinical point of view.

## Acknowledgments

The authors thank all our friends and members of staff of our departments, who encouraged us to complete the study.

## Authors' contributions

The study was planned by EB, FP and NM. The literature review was performed by RN, FP, and EB. MN and FP performed the experiments and drafted the manuscript. RN and FP performed the experimental procedure. The statistical analyses and interpretation of data were carried out by FP. All the authors critically revised the manuscript for intellectual content. All the authors have read and approved the final manuscript.

## Funding

The study was sponsored by the authors. The authors received no funding from any other individual or institution.

## Competing interests

The authors declare no competing interests with regards to the authorship and/or publication of this article.

## Ethics approval

The study was supported by Dental Faculty of Dentistry, Tabriz University of Medical Sciences. The study protocol was approved by the Ethics Committee at Tabriz University of Medical Science (TBZMED: T215).
